# NMR-guided identification of CYP11A1–Adrenodoxin interactions that differentially govern cholesterol and vitamin D3 metabolism

**DOI:** 10.1016/j.jbc.2025.110428

**Published:** 2025-06-26

**Authors:** Janie E. McGlohon, Jacob Logothetis, D. Fernando Estrada

**Affiliations:** Department of Biochemistry, Jacobs School of Medicine and Biomedical Science, University at Buffalo, Buffalo, New York, USA

**Keywords:** adrenodoxin, cholesterol, cholesterol side-chain cleavage enzyme, CYP11A1, NMR, P450scc, vitamin D3

## Abstract

Cytochromes P450 (CYPs) are heme-containing enzymes essential for a range of biochemical processes, including steroidogenesis and vitamin D metabolism. Among mitochondrial CYPs, CYP11A1 catalyzes both cholesterol side-chain cleavage, producing pregnenolone, and hydroxylation of vitamin D3, producing 20(OH)D3. Previous studies have shown that substrates can modulate CYP11A1 protein–protein interactions with the redox partner Adrenodoxin (Adx), but the structural basis of the substrate-specific modulation towards Adx is not known. In this study, we investigated whether there exist contact(s) between CYP11A1 and Adx that are differentially influenced by cholesterol and vitamin D3, and whether these substrate-specific contacts are important for CYP11A1 monooxygenation of vitamin D3 or side chain cleavage of cholesterol. Utilizing 2D NMR spectroscopy in combination with solubilization of substrates with hydroxypropyl-β-cyclodextrin, we were able to isolate M77 of Adx α helix-3 as a substrate-specific contact towards CYP11A1. Site-directed mutagenesis of Adx M77 into M77L and M77S and mutagenesis of the corresponding CYP11A1 contact (W418A) revealed differential effects towards cholesterol and vitamin D3 metabolism. These data suggest that CYP11A1 protein–protein interactions with Adx are uniquely driven by substrate specificity and shed light on potential substrate-sensitive recognition in other mitochondrial CYPs. These findings are further discussed in the context of a modeled interaction between CYP11A1 and the reduced (functional) form of Adx in which Adx M77–CYP11A1 W418 is the driving constraint. Moreover, this study describes an NMR-based protocol that is broadly applicable towards the investigation of other substrate-sensitive CYP–redox partner interactions.

Cytochromes P450 (CYPs) are a superfamily of heme-containing enzymes that, despite sharing a common structural fold, catalyze a diverse range of reactions (1, 2, 3). As such, mammalian CYPs occupy central roles in numerous endogenous metabolic pathways as well as performing early-stage detoxification of xenobiotics (1, 3). Among vertebrates, seven mitochondrial CYPs play crucial roles in reactions involving steroids and vitamins A and D (3, 4, 5). All mitochondrial CYPs require the presence of the iron-sulfur protein Adrenodoxin (Adx) as the sole redox partner to transfer the electrons required to generate the highly reactive iron-oxo species (5). Previous work by our group and by others has shown that the first two steps in the CYP reaction cycle (substrate binding and Adx association) are, to some extent, interdependent and together represent a key regulatory point in CYP-mediated metabolism (6, 7, 8, 9, 10, 11). For example, surface plasmon resonance studies have shown that the affinity of the cholesterol-metabolizing enzyme CYP11A1 interaction with Adx is influenced in a substrate-specific manner; however, the structural basis of substrate-specific modulation towards Adx is not known (11).

In the current study, we employ 2D ^1^H,^15^N-Heteronuclear Single Quantum Coherence (HSQC) NMR to investigate the interaction between Adx and CYP11A1 at the atomic level and in a solution environment. CYP11A1 is ideally suited to investigate substrate-specific effects on the protein complex since it accepts cholesterol and vitamin D3 as substrates for very different reactions (2, 12). Initially identified as P450scc (side-chain cleavage enzyme), CYP11A1 catalyzes the first and rate-limiting step of steroid hormone biosynthesis by converting cholesterol into pregnenolone *via* cleavage of the cholesterol side chain (2, 6, 13). Pregnenolone is the precursor for all steroid hormones and is thus essential for life (14, 15). However, CYP11A1 was more recently confirmed to also metabolize cholecalciferol (vitamin D3) by hydroxylating the carbon-20 position to produce 20(OH)D3, followed by further hydroxylation of the vitamin D3 side chain (16, 17). Notably, downstream 20(OH)D3 metabolites are vitamin D3 receptor agonists and are anti-inflammatory but are less toxic at pharmacological doses than the classical vitamin D3 hormone 1,25(OH)_2_D3 (calcitriol), meaning that CYP11A1 mediates an alternative and potentially therapeutic route of vitamin D3 activation in the body (16, 17, 18, 19, 20, 21, 22).

Understanding how cholesterol and vitamin D3 differentially modulate the CYP11A1–Adx interaction will provide mechanistic insight into the process that regulates Adx preferential transfer of electrons towards one CYP–substrate complex over another. This may be of particular importance in tissues where multiple substrates are available. For example, CYP11A1-mediated metabolism of both cholesterol and vitamin D3 are known to occur in the skin (22, 23, 24). Moreover, mitochondrial CYP27A1, expressed at similar levels as CYP11A1 in the skin, initiates the classical vitamin D3 activation pathway by converting vitamin D3 to 25(OH)D3 (23). This product is subsequently hydroxylated by CYP27B1 to generate the biologically active form, 1,25(OH)_2_D3 (25, 26). While CYP11A1 can alternatively metabolize vitamin D3, it is not known to metabolize 25(OH)D3 (2). Therefore, a portion of the vitamin D3-related CYP11A1 function in the skin consists of the nonclassical vitamin D3 pathway. In tissues where vitamin D3 and cholesterol are both present, and CYP11A1 and CYP27A1 expression levels are comparable, this raises the question whether competition exists for substrates or for reduced Adx. Therefore, we expect that by understanding substrate-specific and CYP-specific interactions, this work sheds light on physiologically relevant competitive states. More broadly, CYP-mediated metabolism in the presence of multiple substrate drugs is a potential basis of drug-drug interactions and drug-induced toxicities.

Our lab has previously used 2D NMR to investigate substrate-induced modulations of Adx with the vitamin D3 inactivating enzyme CYP24A1 (7, 27, 28). However, these prior studies involved a singular substrate (therefore, comparisons between substrates were not possible). Moreover, due to the limited solubility of vitamin D3, the CYP–substrate complex was pre-formed and then added together to ^15^N-labeled Adx. This made it especially challenging to separate substrate-driven effects from effects of the protein-protein interaction.

In this study, we first established a baseline of the bovine CYP11A1–Adx complex in solution without substrate. Until now, no corresponding NMR study has been undertaken to understand the Adx–CYP11A1 interaction. In general, the complex was found to resemble the binding mode of Adx in the co-crystal structure of the human complex (PDB:3N9Y), with the interaction primarily affecting residues on the putative CYP binding site on α helix-3 (2). However, additional peak broadening on Adx α helix-1 was also observed, indicating that solution-binding data are reporting additional interactions that are not reflected in the crystal structure of the complex. Next, to isolate substrate-specific effects on the redox complex, we used a new strategy of first solubilizing cholesterol and vitamin D3 using hydroxypropyl-β cyclodextrin (HPCD), thus enabling the titration of substrates into the pre-formed CYP11A1–Adx complex. These experiments led to the identification of the α helix-3 residue M77 as a candidate for substrate-specific modulation of the protein interface. Site-directed mutagenesis and reconstituted functional assays against both substrates confirmed that the interaction between M77 of Adx and W418 of CYP11A1 differentially governs cholesterol and vitamin D3 metabolism. Another key finding from this work is that the Adx mutation M77S selectively enhances vitamin D3 metabolism, despite disorder at the α helix-3 CYP recognition site. Together, these findings inform a revised model of the CYP11A1–Adx protein complex in solution and with reduced Adx and provide insight toward an important interfacial substrate-specific contact for other mitochondrial CYPs.

## Results

### Solution binding of CYP11A1 induced NMR peak broadening in **α** helix-1 and **α** helix-3 of ^15^N-Adx

Prior to measurement of substrate-induced changes in the CYP11A1 complex with Adx, it was first necessary to establish a baseline for the protein interaction in solution and in the absence of substrate. ^1^H,^15^N-HSQC spectra were acquired of ^15^N-Adx alone and in the presence of increasing concentrations of CYP11A1. As reported previously with CYP24A1 (28), these samples were prepared using a two-step buffer exchange in which the CYP is first exchanged into and washed with a high salt, detergent-free buffer, then combined with ^15^N-Adx prior to exchange into the final NMR buffer, which is at a lower ionic strength (see Experimental Procedures for a detailed protocol). This buffer exchange sequence ensures that the CYP remains stable by always maintaining an excess of detergent, high concentrations of NaCl, or Adx, with the final sample consisting of the CYP11A1–^15^N-Adx complex in a low salt buffer (50 mM NaCl) and with extraneous CHAPS detergent having been removed in a previous step.

**Figure 1:**
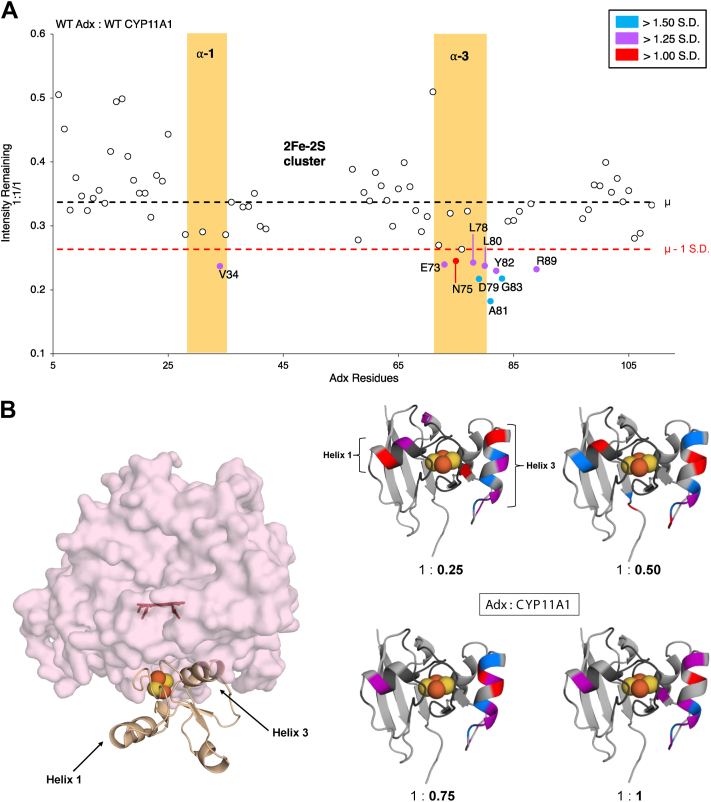
**Titration of WT CYP11A1 against ^15^N-WT Adx.***A*, 2D NMR scatterplot showing ^15^N-Adx peak broadening for all assigned residues in the presence of equal molar CYP11A1. Amide peaks near the 2Fe-2S cluster are not observable. The intensity plot illustrates the average (*μ*) Adx peak intensity remaining (----, *black*) resulting from the interaction with CYP11A1. A cutoff of 1 standard deviation (----, *red*) away from the average (*μ* – 1 S.D.) was applied to identify residues that undergo peak broadening relative to the overall assigned peaks. *B*, the crystallographic complex of the CYP11A1 (surface, *light pink*) and Adx (cartoon, wheat) complex (PDB: 3N9Y) is shown (*left*). Differentially affected residues are mapped onto the structure of Adx (PDB: 1CJE) upon titration of CYP11A1 (*right*), marked by a loss of peak intensity ratio greater than 1.50 (*blue*), 1.25 (*purple*), or 1.0 (*red*) standard deviation away from the average remaining intensities. Unassigned peaks are mapped on the structure of Adx (*black*).

As expected, the titration with CYP11A1 resulted in broadening of the ^15^N-Adx NMR signal. Approximately 81% to 33% of the original signal intensity remained upon the addition of 0.25 and 1.0 equivalents of CYP11A1, respectively (Fig. 1*A*) (Full-intensity plots shown in Supporting Information Fig. S1). An analysis of individual residues was carried out by comparing a peak intensity ratio that represents the intensity remaining from the original (unbound) peak; residues displaying more than one standard deviation from the mean were identified as significant. Moreover, to analyze binding at the core folded domain of Adx, the intensities corresponding to the C-terminal tail residues (110–128) were removed from the analyses. Most of the C-terminal tail residues did not display significant peak broadening in the current data and were demonstrated in a recent study as unnecessary for binding to the CYP (28). Moreover, the high intensity remaining in these residues masked (statistically) the differential effects observed in the core CYP-binding domain of Adx.

Increasing CYP11A1 resulted in focused peak broadening centered on α helices-1 and -3 of ^15^N-Adx. Residues on or near the ^15^N-Adx α helix-3 that were differentially affected (marked by a loss of peak intensity ratio greater than 1.50, 1.25, or 1.0 standard deviations) consisted of E73, N75, L78, D79, L80, A81, Y82, and G83 (Fig. 1), with D79, A81, and G83 being the most affected at the final equal molar titration with CYP11A1. Interestingly, while the effects at the CYP recognition site on α helix-3 were expected (there have been extensive previous structural and mutagenesis studies that confirm α helix-3 as the CYP binding site) (2, 7, 9, 27, 29, 30, 31, 32, 33, 34), this analysis also revealed differential peak broadening near α helix-1. This was particularly noticeable at the lower titration points; α helix-1 residues D31 and V34 demonstrated disproportionate peak broadening, but with increasing amounts of CYP11A1, this effect focused on V34 (Fig. 1*B*). Notably, α helix-1 does not form part of the CYP–Adx interface in the crystal structure of the complex (PDB:3N9Y), thus indicating that solution NMR is accessing additional and likely secondary interactions (Fig. 1*B*).

### 2D NMR spectra report unique cholesterol and vitamin D3-induced modulations at the CYP11A1–Adx interface

**Figure 2:**
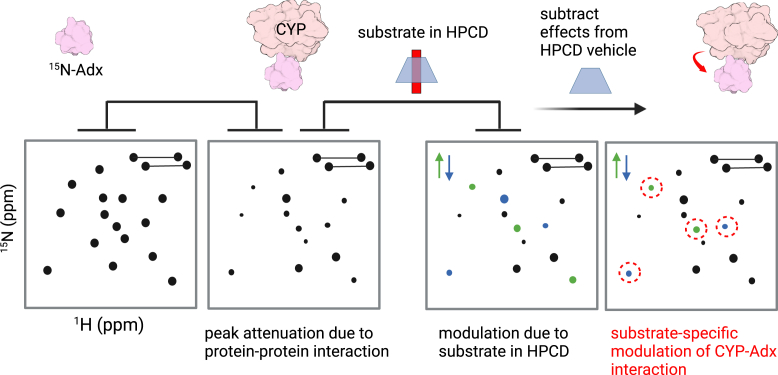
**2D NMR experimental workflow to identify substrate-induced CYP-Adx effects.** The first panel (*left*) shows a representation of the 2D NMR spectrum of ^15^N-labeled Adx alone, serving as the baseline reference. Addition of CYP leads to peak attenuation due to the protein interaction. The third panel includes the addition of the substrate-HPCD complex. Nonspecific interactions between HPCD and ^15^N-Adx are subtracted in the final step. The final panel highlights substrate-specific modulations, with distinct changes in peak intensities and shifts (indicated by *green* and *blue arrows*) revealing substrate-induced effects on the CYP-Adx interaction (*red dashed circles*).

Since the presence of cholesterol is known to promote binding between CYP11A1 and Adx (11), 2D NMR was then used to investigate the ternary ^15^N-Adx–CYP11A1–substrate complex in solution. However, rather than titrate the substrate-bound enzyme into samples of ^15^N-Adx, as has been done previously with a different CYP (34), the ^15^N-Adx–CYP11A1 complex was first pre-formed without substrate, and the effect of substrate was measured in subsequent samples that contained increasing concentrations of cholesterol or vitamin D3 that had been solubilized in a solution of 25% HPCD. Delivery of the HPCD-bound substrate to CYP11A1 was separately confirmed using absorbance difference assays, as described later in this study. This experimental workflow is summarized in Figure 2. In brief, by first establishing the baseline effects of the protein-protein interaction, peak attenuation that results from the CYP11A1 interaction with Adx can be separated from changes in the NMR spectra that result solely from the presence of the substrate. Moreover, HPCD encapsulation allows for the controlled delivery of high concentrations of substrates that are otherwise poorly soluble in aqueous buffer. This approach has previously been used for CYP11A1 binding and functional assays (35, 36, 37), and cyclodextrins have been used previously to facilitate NMR experiments (38). The contributions of empty HPCD first had to be established. Addition of HPCD without substrate was found to result in modest changes in select peak intensities, likely due to non-specific interactions between HPCD and ^15^N-Adx. The HPCD-affected residues in the control experiment are plotted in Supporting Information Figure S2*A*. Many of these same residues (for example, decreased intensities at V9, T18, T21, and I25) are also affected in all substrate-bound data. However, since this effect could not be separated from contributions of the empty HPCD vehicle, changes in these residues were monitored but were not interpreted to be solely derived from substrate-specific modulations.

**Figure 3:**
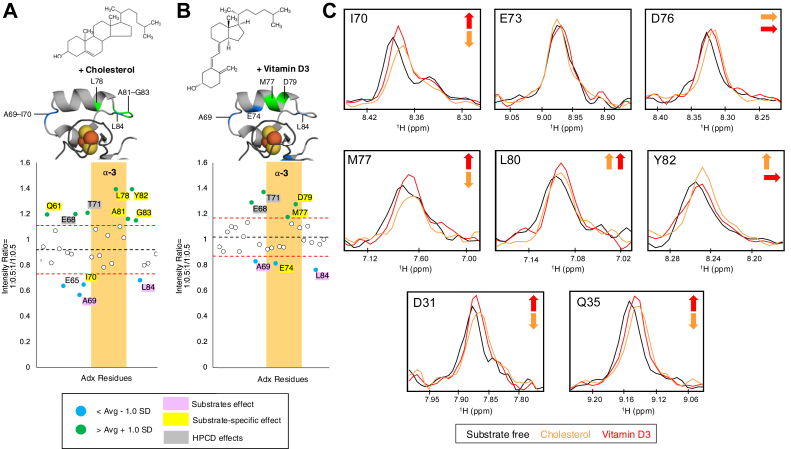
**Substrate-induced effects on the ^15^N-Adx–CYP11A1 complex 2D NMR spectra with cholesterol and vitamin D3.** The intensity plot showing effects of HPCD (*gray*), substrates (*pink*), and substrate specific (*yellow*) on the ^15^N-Adx α helix-3 region with CYP11A1 in the presence of (*A*) cholesterol and (*B*) vitamin D3. The intensity plot illustrates the average Adx peak intensity remaining (----, *black*) resulting from the interaction with CYP11A1 and substrates. A cutoff of 1 standard deviation (----, *red*) away from the average was applied to identify residues that undergo peak broadening relative to the overall assigned peaks. Differentially affected residues are mapped onto the structure of Adx ⍺ helix-3 (PDB: 1CJE) upon titration of CYP11A1, marked by a decrease (*blue*) or increase (*green*) of peak intensity ratios greater than 1.0 standard deviation away from the average remaining intensities. *C*, one-dimensional slices of the ^1^H dimension highlighting substrate-induced effects on the intensities of α helix-3 residues I70, E73, D76, M77, L80, Y82, and α helix-1 residues D31 and Q35.

Cholesterol and vitamin D3 titrations were then acquired against a ^15^N-Adx–CYP11A1 protein ratio of 1:0.5. A lower concentration of CYP11A1 was chosen to both lessen the overall extent of peak attenuation and to ensure an excess of substrate relative to CYP by the end of the titration (1:0.5:1). A comparison of the highest titration points (1:0.5:1) for cholesterol and vitamin D3 are shown in Figure 3, *A* and *B*, while intensity plots for the full titration are included in Supporting Information Figure S2. Interestingly, the addition of substrate resulted in significant increases or decreases in peak intensities on or near the putative CYP binding surface of α helix-3. Residues that were modulated by the presence of a specific substrate or by both substrates (and were not affected by the presence of HPCD alone) are highlighted in yellow and in light pink, respectively. Cholesterol was found to induce a unique increase in the intensities of L78 of α helix-3, as well as A81, Y82, and G83 on the loop succeeding α helix-3. Cholesterol was also found to induce additional peak broadening at I70 preceding α helix-3. The cholesterol-specific effects are mapped onto α helix-3 of Adx in Figure 3*A*.

In contrast, vitamin D3 caused a unique increase in intensity at residues M77 and D79 of α helix-3 and a decrease in E74, while having no significant effect on the α helix-3 loop residues A81-G83 (Fig. 3*B*). The panel in Figure 3*C* shows an overlay of 1D slices of the ^1^H dimension from α helix-1 residues D31 and Q35, α helix-3 residues E73, D76, M77, L80, and the loop residues I70 and Y82. The observation that this pattern was distinct for M77 as compared to the nearby loop residue Y82 indicates that this region undergoes a localized effect in response to each substrate. While the most pronounced effects involved α helix-3, other affected regions included the α helix-1 residues Q35 and D31, which exhibited opposite modulations with cholesterol and vitamin D3, respectively, as well as α helix-2 residue Q61, which increased in intensity only in the presence of cholesterol (Supporting Information Fig. S2).

Finally, to confirm that the subtle nature of the changes in peak intensities is consistent, one more experiment was acquired using an equal molar ratio of CYP11A1–Adx with cholesterol (1:1:1) (Supporting Information Fig. S3). Notably, the pattern of differential peak changes was preserved at these higher CYP11A1 ratios (compare to Fig. 3*A*). Specifically, the decrease in M77 was also present. However, due to the low amount of signal remaining at these ratios (approximately 11% of the original intensity remains in these spectra) and the larger resources required for sample preparation, subsequent titrations were performed using a reduced CYP11A1 concentration of 1:0.5:1 (Adx:CYP11A1:Substrate).

### CYP11A1 vitamin D3 and cholesterol metabolism are altered in response to Adx **α** helix-3 M77 mutations

One of the Adx α helix-3 residues that was uniquely affected by titration of substrates into NMR samples of the complex with CYP11A1 was M77. ^15^N-Adx M77 is in the middle of α helix-3 and demonstrated a decrease in intensity in spectra of the ternary complex with cholesterol, but increased intensity when vitamin D3 was present (Fig. 3*C*). To our knowledge, there have been no reports demonstrating that M77 plays an active role in the protein-protein interaction with CYP11A1 or with any other mitochondrial CYP, although the crystallographic interface indicates a possible interaction with the side chain of W418 of CYP11A1 (2).

To determine whether the NMR data indicate a CYP11A1 substrate-specific or substrate-driven effect through Adx M77, this position was mutated to either a serine or a leucine. These mutations were designed to assess whether Adx M77 interacts with the CYP11A1 proximal residue W418 through a methionine-aromatic interaction (39) vs a simple hydrophobic interaction (M77L), or whether replacement of the nonpolar side chain entirely would disrupt function (M77S).

**Figure 4:**
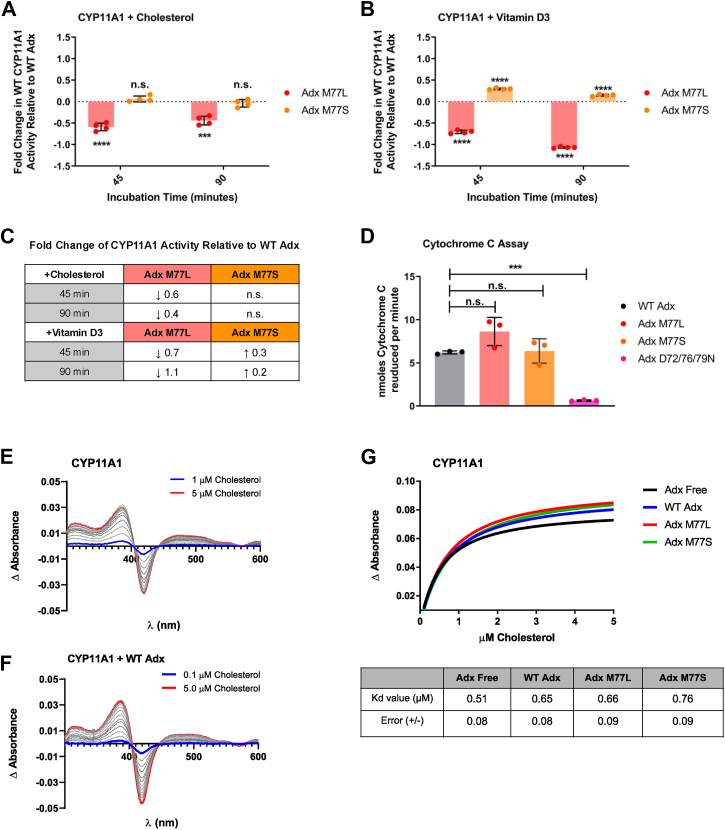
**Evaluating the effects of Adx M77 mutations on CYP11A1 function.** Reconstituted CYP11A1 functional assays measuring the fold change in CYP11A1 metabolism of (*A*) cholesterol and (*B*) vitamin D3 with Adx M77L (*red*) and M77S (*orange*) relative to WT Adx over 45 and 90 min reaction times. *C*, a summary presenting the fold changes in CYP11A1 activity. *D*, cytochrome C assays indirectly measuring the reduction of Adx WT (*gray*), M77L (*red*), and M77S (*orange*) by AdR, with the Adx ⍺ helix-3 triple mutant D72N/D76N/D79N (*pink*) as a negative control. Difference spectra of cholesterol solubilized with HPCD are shown for CYP11A1 and CYP11A1 in the presence of excess WT Adx in (*E*) and (*F*), respectively. *G*, the binding curve of cholesterol binding to CYP11A1 in the absence of Adx (*black*), excess WT Adx (*blue*), Adx M77L (*red*), or Adx M77S (*green*). Kd values derived from triplicate binding assays are also summarized below. Full saturation plots are summarized in Supporting Information Figure S3. All functional assays were carried out in quadruplicate and significance determined using one-way ANOVA with an α of 0.05. Significance is indicated as (∗∗∗∗ for *p*-values < 0.0001). Tukey’s test was used for *post hoc* ANOVA analysis.

Activity of CYP11A1 was measured using either 25% HPCD-cholesterol or -vitamin D3 as substrate. To better compare pregnenolone formation and vitamin D3 depletion, activity data are represented as fold changes in activity relative to the native condition, as described further under Experimental Procedures. Reconstituted CYP11A1 functional assays measuring the side chain cleavage of cholesterol in the presence of Adx M77L resulted in an average decrease by 0.6 fold in the amount of pregnenolone produced compared to WT Adx (at 45 min) (Fig. 4*A*). Adx M77L also resulted in an average decrease by 0.7 fold in vitamin D3 depletion over the same incubation time (Fig. 4*B*). Figure 4*C* summarizes the fold change in CYP11A1 activity in response to Adx M77S and M77L. Interestingly, in the presence of the least chemically conservative mutation, Adx M77S, CYP11A1 production of pregnenolone was comparable to that from WT Adx, whereas vitamin D3 metabolism was enhanced by approximately 0.3-fold (at 45 min).

Next, the ability of Adx mutants to alter binding of CYP11A1 substrates was examined. In CYP enzymes, the substrate binds to the distal side of the heme, displacing a water molecule and positioning itself for catalysis, while Adx binds to the proximal side, transferring electrons to the heme for catalysis, which is coordinated to the CYP enzyme *via* a cysteine-thiolate bond (2, 6). In brief, substrate binding to the active site of cytochrome P450 enzymes is typically monitored by changes in the UV-Visible absorbance spectrum of the heme group. This occurs because substrate binding displaces the water molecule coordinated to the heme iron in the resting state. For CYP11A1, this results in a spectral shift from ∼418 nm (substrate-free) to the ∼390 nm region (substrate-bound). The presence of Adx has been documented to promote the CYP–substrate interaction as indicated by an increase in the net absorbance changes that occur (8, 9, 10).

Spectral binding assays were conducted with CYP11A1 and 25%HPCD-cholesterol and -vitamin D3, in the absence and presence of a 10-fold excess of WT Adx, M77L, and M77S (Supporting Information Fig. S4). Titration of empty 25% HPCD against CYP11A1 showed no spectral changes (data not shown). As reported previously (2), vitamin D3 produced a weak spectral response, even in the presence of Adx, so titration data were quantifiable only for cholesterol (data not shown). As expected, titrating cholesterol against CYP11A1 resulted in a type-I shift of the Soret peak, resulting in a difference spectrum indicating an increase near 385 nm and a decrease near 418 nm (Fig. 4*E*). The addition of excess WT Adx did not significantly alter the cholesterol dissociation constant (Kd) but caused a modest increase in the net absorbance (Fig. 4, *F* and *G*). Here, the addition of excess Adx M77L and M77S showed no significant differences compared to WT Adx. Together, these data suggest that M77 mutations of Adx do not alter the recognition of cholesterol and that the differences in CYP11A1 cholesterol metabolism between WT and M77L are not due to disruption of substrate binding, at least as measured by changes in heme absorbance.

Finally, to ensure that the observed significant changes in CYP11A1 substrate metabolism are not due to a disruption in the recognition between Adx and Adrenodoxin reductase (AdR), cytochrome C assays were carried out to indirectly observe the reduction of Adx by AdR. No significant difference was observed between WT Adx and AdR electron transfer compared to Adx M77L and Adx M77S (Fig. 4*D*). The triple mutant Adx D72N/D76N/D79N was used as a negative control since these Adx α helix-3 negatively charged aspartic acid residues are known to interact with AdR and the proximal surface of mitochondrial CYPs (2, 28). These data suggest that the M77 mutants do not disrupt the electron transfer between AdR and Adx, and therefore, differences in CYP11A1 activity are a result of changes in the substrate-CYP-Adx ternary complex.

### M77 of Adx and W418 of CYP11A1 represent a substrate-driven contact

Next, we wanted to elucidate the importance of a possible corresponding Adx M77 contact on the CYP11A1 proximal surface. The crystal structure of the human CYP11A1-Adx complex positions the Adx α helix-3 residue L80 as forming a likely nonpolar interaction approximately 3Å from the CYP11A1 residue W418 (PDB:3N9Y) (2), while the sidechain of M77 is shown farther away at 8Å. However, it should be noted that the positioning of M77 and L80 is likely to change when Adx is in its reduced conformation (40). Given that Adx M77L significantly disrupted CYP11A1 metabolism of both cholesterol and vitamin D3, we introduced a W418A mutation in CYP11A1 to minimize the bulk of the hydrophobic side chain of tryptophan while retaining a nonpolar residue. The CYP11A1 W418A mutation was found to produce a stable complex with carbon monoxide and bound cholesterol similar to WT.

**Figure 5:**
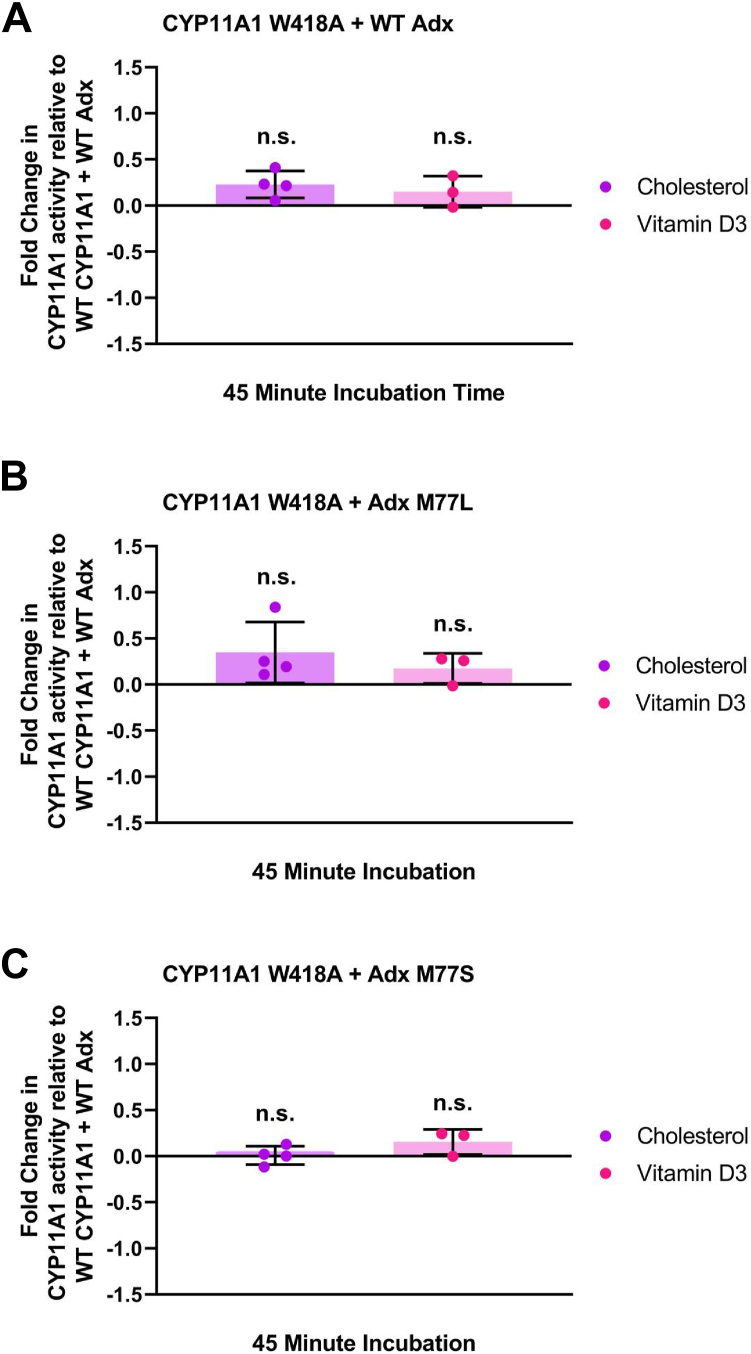
**Evaluating the effects of CYP11A1 W418A on substrate metabolism in the presence of Adx WT, M77L, and M77S.** CYP11A1 activity was measured as fold change relative to the WT CYP11A1–WT Adx complex activity with cholesterol (*purple*) or vitamin D3 (*pink*). Changes in CYP11A1 W418A activity towards cholesterol and vitamin D3 are in the presence of (*A*) Adx WT, (*B*) M77L, and (*C*) M77S. Significance for assay results was determined using one-way ANOVA with an α of 0.05. All *p*-values were above 0.05. Dunnett’s test was used for *post hoc* ANOVA analysis.

Notably, relative to WT CYP11A1, W418A did not disrupt either vitamin D3 hydroxylation or the side chain cleavage reaction of cholesterol (Fig. 5*A*). However, whereas Adx M77L caused a significant disruption of WT CYP11A1 activity (Fig. 4*A*), its combination with W418A restored CYP11A1 function (Fig. 5*B*). In contrast, the addition of CYP11A1 W418A with Adx M77S resulted in no significant changes in activity with vitamin D3, compared to WT, effectively nullifying increased activity due to M77S alone (Fig. 5*C*). These findings demonstrate not only that this specific interaction is a regulatory feature of CYP11A1 function, but that changes at the protein interface differentially affect the specific cholesterol and vitamin D3 reactions.

### M77S remodels the CYP recognition site of Adx

**Figure 6:**
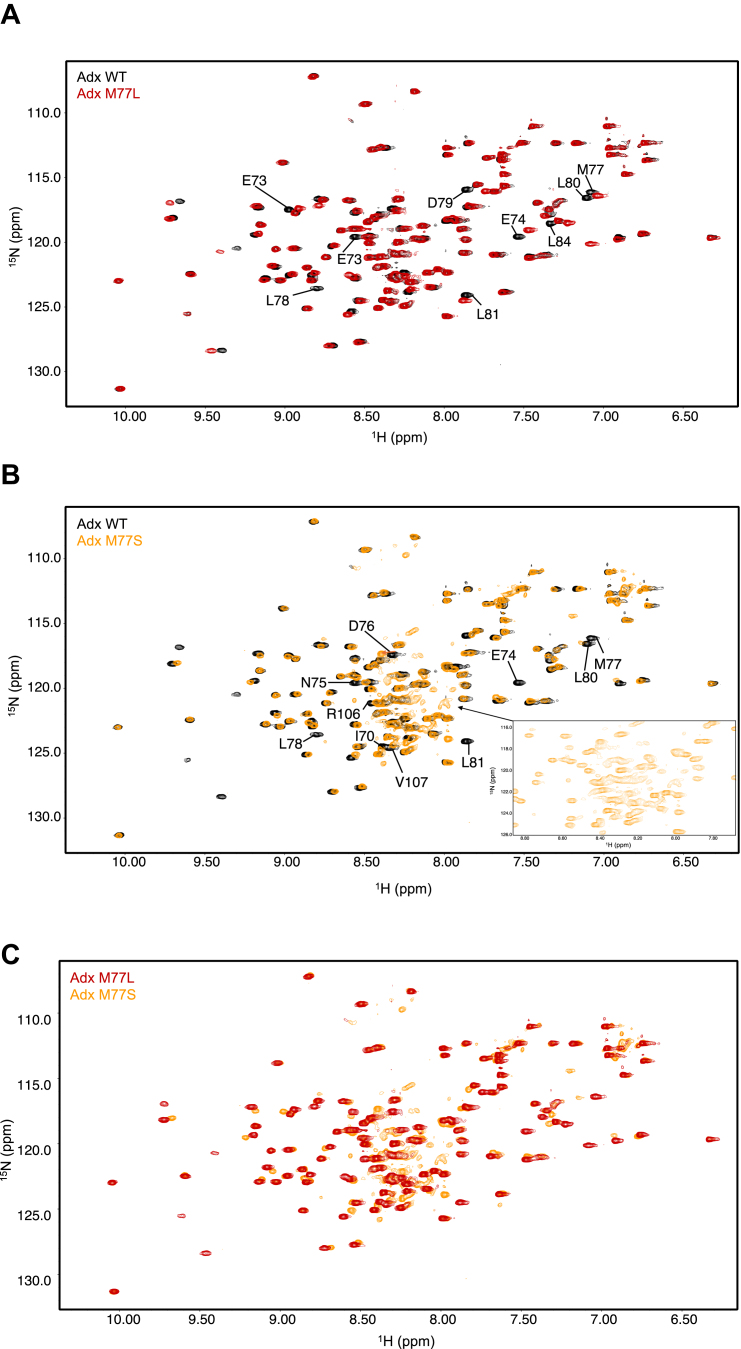
**2D ^1^H,^15^N-HSQC overlay of ^15^N-Adx M77 mutants with ^15^N-WT Adx.** Overlay of (*A*) Adx M77L (*red*) overlaid with WT (*black*), (*B*) Adx M77S (*orange*) overlaid with WT (*black*), and (*C*) Adx M77L (*red*) overlaid with M77S (*orange*). Peak perturbations due to Adx M77L or Adx M77S mutations relative to WT Adx are indicated in *black* text.

When compared to WT Adx, Adx M77L significantly reduced WT CYP11A1 activity towards vitamin D3 and cholesterol, while Adx M77S had no significant effect on cholesterol metabolism but significantly enhanced activity against vitamin D3 (Fig. 4). To determine the structural effects at the site of CYP recognition, the ^1^H,^15^N-HSQC spectra of Adx M77L and M77S were acquired and compared to the spectrum of WT Adx. Both mutations resulted in the residues in the α helix-3 region exhibiting peak perturbations (Fig. 6). However, while perturbations resulting from M77L appear to be localized near the site of mutation, and the peak distribution for this mutant generally resembled that of WT, more noticeable changes in the spectrum of M77S suggest a change in the Adx fold (Fig. 6). Importantly, the apparent loss of peaks at certain residues, combined with the appearance of new peaks near the center of the spectrum, indicates that M77S likely also induces partial disorder in the Adx fold, specifically affecting the structure of ⍺ helix-3 (Fig. 6*B*).

### Adx M77L and M77S differentially modulate the substrate-bound CYP11A1–Adx ternary complex

**Figure 7:**
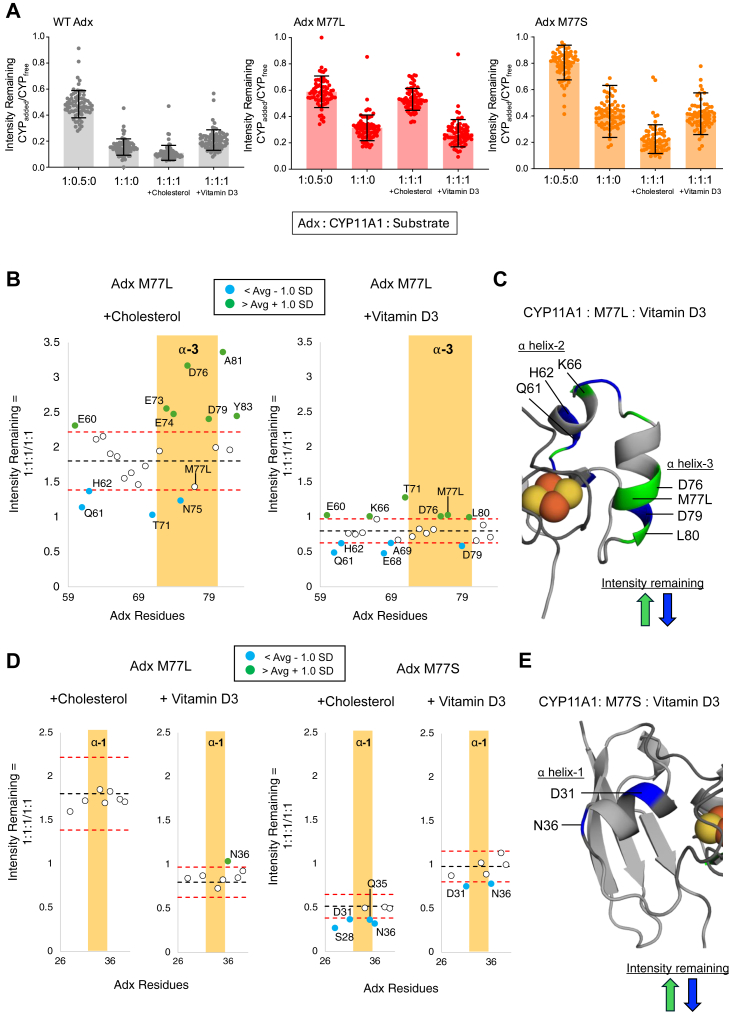
**2D NMR analysis of substrate-driven effects on Adx M77 mutants.***A*, overall effect of the substrate–CYP11A1 complex on Adx. Average per residue remaining intensities are shown for WT Adx (*black*), Adx M77L (*red*), and Adx M77S (*orange*) with 0.5 and equal molar of CYP11A1, and in the presence of equal molar CYP11A1 and substrates cholesterol or vitamin D3. Standard deviation bars are shown in black for all conditions. *B*, intensity scatterplot of substrate-driven effects on the α helix-3 region of Adx M77L with cholesterol (*left*) and vitamin D3 (*right*). *C*, the affected regions of Adx M77L from the CYP11A1–vitamin D3 complex are mapped onto the structure of Adx. *D*, intensity scatterplot of substrate-driven effects on the α helix-1 region of Adx M77L (*left*) and M77S (*right*). (*E*, the substrate-driven effects of Adx M77S α helix-1 with the vitamin D3CYP11A1 complex are mapped onto the structure of Adx. The data in panel (*A*) represent individual amide resonance peak intensities and do not represent replicate data. Therefore, these data were not evaluated for statistical significance.

Next, to determine how M77L and M77S affect the interaction with CYP11A1, the ^1^H,^15^N-HSQC spectra of each mutant were acquired in the presence of the enzyme. It should be noted that due to multiple mutation-induced peak perturbations, some residues were not monitored (for example, L78 in the case of M77L, and I70, E74, D76, R106, and V107 in the case of M77S). The addition of CYP11A1 to Adx M77L and M77S at a 0.5:1 and 1:1 ratios resulted in a decrease in the average per-residue peak intensities, suggesting that these mutations do not prevent CYP11A1 association with Adx (Fig. 7, *A* and *B*).

Next, the substrate-affected regions were mapped onto the Adx structure. Both substrate-bound M77L complexes resulted in increased peak intensities for residues near or on ⍺ helix-3 (Fig. 7*C*), while M77L with vitamin D3 also showed a significant decrease in the peak intensities for residues near ⍺ helix-2 (Fig. 7*C*, mapped in Fig. 7*D*). These maps indicate that the substrate-driven effect with M77L may result in a repositioning of Adx at the CYP11A1 proximal surface that negatively affects the function of the complex.

Mapping the differential NMR intensity data for the M77S experiments was more challenging, since a number of residues, particularly near ⍺ helix-3, undergo chemical shift perturbations (Fig. 6). However, a consistent pattern in these experiments was that Adx M77S in the presence of cholesterol showed differential line broadening in α helix-1 residues S28, D31, Q35, and N36 (Fig. 7*D*). A similar effect on ⍺ helix-1 was observed with vitamin D3 (Fig. 7*D*, mapped in Fig. 7*E*). In contrast, this effect was not observed at all for the complex with M77L. It should be noted that involvement of ⍺ helix-1 was previously observed upon addition of cholesterol to the native complex (previous Supporting Information Fig. S2) and to some extent with the substrate-free complex (Fig. 1). This likely correlates with the ability of M77S to preserve or enhance catalytic function, possibly by compensating the structural changes occurring in ⍺ helix-3 with supplementary contacts in ⍺ helix-1.

### Adx M77 is a mitochondrial CYP-specific contact

**Figure 8:**
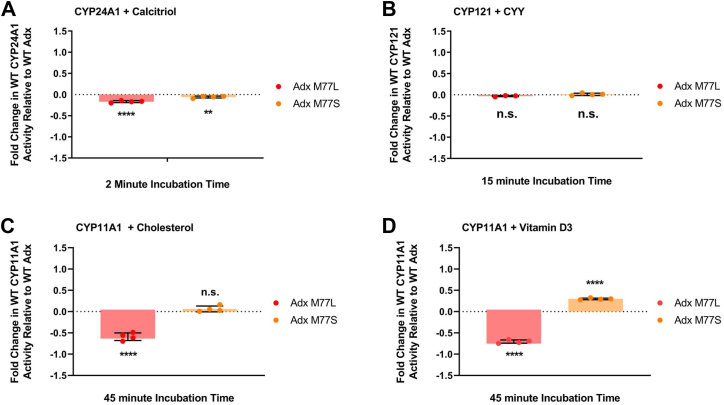
**Measuring the impact of Adx M77 mutants on CYP24A1 and CYP121 activity.** Reconstituted functional assays measuring the fold change of CYP substrate metabolism in the presence of Adx M77L (*red*) and M77S (*orange*) relative to WT Adx, with (*A*) CYP24A1 hydroxylation of calcitriol, (*B*) CYP121 phenol coupling reaction of CYY. For comparison, the effects of each mutant on CYP11A1 activity (*C*) and (*D*), are reproduced from Figure 4. Significance for assay results was determined using one-way ANOVA with an α of 0.05. Significance is indicated as (∗∗∗∗ for *p*-values < 0.0001) and (∗∗ for *p*-values = 0.0032). Dunnett’s test was used for *post hoc* ANOVA analysis.

We then asked whether the ability of M77S and M77L to modulate CYP function was limited to CYP11A1, or whether this represented a more widespread substrate-driven contact site. The Adx mutants were incorporated into reconstituted assays using different CYP enzymes that share little sequence homology with CYP11A1. Mitochondrial CYP24A1 (rat) is a vitamin D3 carbon-24 hydroxylase and features an isoleucine in place of W418 of CYP11A1 (41, 42). The bacterial enzyme CYP121 from *Mycobacterium tuberculosis* performs a phenol ring coupling reaction of dicyclotyrosine (cYY) and features an arginine in this position (43). Interestingly, Adx M77L and M77S had a very minor negative impacted on CYP24A1 activity towards calcitriol and no change on CYP121 metabolism of cYY (Fig. 8, *A* and *B*). This suggests that while M77 may be an active protein contact for mitochondrial CYPs, it is particularly important for CYP11A1 activity (Fig. 8, *C* and *D*).

### Modeling of the Adx M77–CYP11A1 W418 contact

**Figure 9:**
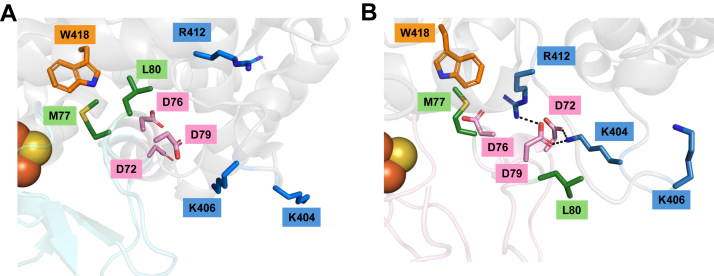
**Docking analysis of CYP11A1 and Adx protein-protein interaction with active residues CYP11A1 W418–Adx M77.***A*, crystal structure of human CYP11A1 with human Adx (PDB: 3N9Y) and (*B*) the highest scoring HADDOCK version 2.4 model of bovine CYP11A1 (PDB:3MZS) with reduced bovine Adx (PDB: 1L6V). Amino acids undergoing structural differences at the interface are indicated and include W418 in *orange* with nonpolar Adx residues in *green*, acidic Adx residues in *pink* and basic CYP11A1 residues in *blue*.

Next, docking analysis was performed to explore the structural implications of the Adx M77–CYP11A1 W418 interaction. Moreover, to correlate the docked complex with functional data, docking was carried out using the NMR-derived structure of reduced bovine Adx (PDB:1L6V) and the crystal structure of bovine CYP11A1 (PDB: 3MZS). This model was then compared to the crystallographic interface of the human CYP11A1–Adx complex (PDB: 3N9Y). To guide the model, residues W418 in CYP11A1 and M77 in Adx were identified as an active interaction in HADDOCK 2.4 (44, 45). A model was selected from 136 structures across 11 clusters. Cluster 2 was identified as having the most favorable HADDOCK score based on van der Waals interactions, electrostatic forces, and structural properties (*e.g.* buried surface area and interfacial RMSD). There were several notable differences in the modeled complex (Fig. 9). For example, the crystal structure positions Adx L80 approximately 3 Å and M77 approximately 8 Å from CYP11A1 W418, whereas the docked structure positions Adx L80 away from the interface and approximately 14 Å away from W418. This suggests that in the reduced form, M77 and L80 are unlikely to form simultaneous contacts with CYP11A1. Moreover, the tryptophan-methionine contact combined with the absence of significant secondary structure (in the reduced form) at the former ⍺ helix-3 allows D72 and D79 of Adx to share an environment with R412 and K404 of CYP11A1. Of note, K404 has previously been identified as an important site of redox partner binding in functional assays (32), despite its orientation away from the interface in the crystallographic structure. Therefore, the modeled complex guided by the substrate-sensitive contact between CYP11A1 W418 and Adx M77 provides new insight into the structure of the complex with reduced Adx, which has been challenging to capture using traditional structural methods.

## Discussion

All seven mitochondrial CYPs must 1) bind a substrate and 2) associate with the redox partner Adrenodoxin (Adx). As mentioned previously, studies have shown these first two steps in the CYP catalytic cycle are a key point in regulating CYP-mediated metabolism. Our lab has previously used NMR spectroscopy to describe substrate-induced modulation of the interaction between Adx and CYP24A1 (34). However, until now no corresponding study had been undertaken to understand Adx and CYP11A1 interactions. Moreover, no NMR study had been undertaken in which multiple and potentially competing substrates were involved. In this work, we demonstrate that 1) 2D NMR can be adapted to isolate subtle substrate-driven changes in the CYP–Adx interface, 2) cholesterol and vitamin D3 uniquely modulate the CYP11A1–Adx interface, inducing distinct substrate-specific changes centered around α helix-1 and -3, 3) Adx residue M77 is a substrate-driven contact, as mutations at this position (M77L and M77S) differentially alter CYP11A1 metabolism of cholesterol and vitamin D3 without disrupting electron transfer from AdR to Adx or substrate binding to CYP11A1, 4) despite its orientation away from the interface in the co-crystal structure, α helix-1 contacts are important as Adx M77L reduces CYP11A1 activity and loses α helix-1 contacts, whereas Adx M77S promotes α helix-1 contacts and does not impact or enhances CYP11A1 activity, and 5) docking analysis using reduced bovine Adx and CYP11A1, guided by the CYP11A1 W418 and Adx M77 interaction, reveals a model that differs from the crystallographic human complex and suggests that, in the reduced form, Adx L80 is unlikely to form a contact with CYP11A1 W418 but includes new interactions between Adx D72/D79 and CYP11A1 R412/K404. These key findings are discussed in more detail below.

The CYP11A1 substrates vitamin D3 and cholesterol are both derive from 7-dehydrocholesterol, in which cholesterol is produced by the reduction of the carbon C7-8 bond and vitamin D3 is formed by breakage of the carbon C9-10 bond on the B ring (12, 46, 47). These structural differences in substrates are predicted to significantly alter the interaction within the CYP11A1 active site, which were expected to result in unique structural changes in the proximal surface (the Adx recognition site) (2). There is previous evidence indicating that CYP11A1 protein−protein interactions with Adx are influenced in a substrate-specific manner (11). While these studies did not include vitamin D3, they do demonstrate substrate-induced modulation of the interaction between CYP11A1 and Adx. For example, cholesterol binding to CYP11A1 significantly increased the affinity of CYP11A1 for Adx by inducing a faster association of the complex, while its product pregnenolone significantly decreased the affinity of the complex by reducing the CYP11A1 rate of association to Adx (11). However, the structural basis of the substrate-specific modulation towards Adx is not known.

In this study, we set out to establish whether there exist contact(s) between CYP11A1 and Adx that are differentially influenced by particular substrates and whether these substrate-specific contacts are important for CYP11A1 monooxygenation of vitamin D3 or side chain cleavage of cholesterol. 2D NMR and uniform ^15^N-labeling of Adx allowed us to observe interactions that were both consistent with the crystallographic structure of the human CYP11A1 fused to Adx (2), as well as unique CYP11A1 substrate-induced effects on the Adx protein. Ligand-free CYP11A1 was found to induce differential peak broadening focused to Adx α helices-1 and -3, with more pronounced peak broadening towards the expected site of interaction at α helix-3. Although α helix-1 contacts are not evident from crystal structures of CYP11A1 in complex with Adx (nor from a co-crystal structure with CYP11B2 (9)), these additional interactions are captured in solution and are consistent with interactions observed between Adx and CYP24A1 (28).

The solubilization of substrates into HPCD allowed us to isolate specific effects driven by the presence of each substrate from changes in the NMR spectra that result only from the protein-protein interaction. Cholesterol and vitamin D3 were both found to modulate residues on Adx α helices-1 and -3 but in different ways. Cholesterol uniquely reduced line broadening at residues L78 and A81-Y82, suggesting loosening of the localized interaction at the loop region following α helix-3. Additionally, cholesterol promoted line broadening towards α helix-1 residue Q35 and α helix-3 residue M77, suggesting a strengthening of the localized interaction at α helices-1 and -3. Conversely, vitamin D3 had the opposite effect at M77 and on the loop region following α helix-3, as well as α helix-1 residue D31. Despite these differences, it should be noted that the peak intensity at L80—which the co-crystal structure indicates interacts with W418 of CYP11A1—is similar with cholesterol as it is with vitamin D3. This suggests that the observed effects on M77 and the adjacent loop region reflect a substrate-specific, localized modulation. Moreover, these differences between vitamin D3 and cholesterol suggest that cholesterol stabilizes interactions near the Adx α helix-3 loop, which promotes a focused effect of Adx residues on α helix-1 and -3 toward the CYP11A1 proximal surface. In part, these differences may explain why CYP11A1 displays lower relative activity towards vitamin D3 (2, 12).

The Adx residue M77 was the only uncharged residue on α helix-3 that was uniquely affected by addition of substrates in the NMR data (Fig. 3). However, M77 has not previously been implicated in a direct interaction with CYP11A1 or with other mitochondrial CYPs. An examination of structures of Adx in complex with human CYP11A1 suggest that L80, rather than M77 forms a nonpolar interaction with W418. Interestingly, replacement of this residue with another (albeit stronger) nonpolar side chain (M77L) resulted in reduced metabolism of both vitamin D3 and cholesterol, with a greater effect towards the latter substrate. Notably, the substrate-bound NMR data revealed that Adx M77L resulted in a loss of contacts towards α helix-1 and increased line broadening towards α helix-2 (Fig. 7, *C*–*E*). Since reduced Adx associates with CYP11A1 proximal surface, we want to highlight that superposition of reduced Adx (PDB:1L6V) with oxidized Adx (PDB:3N9Y) in the co-crystal structure of human CYP11A1(PDB:3N9Y) positions Adx L80 toward the meander region of CYP11A1 and instead positions Adx M77 toward the proximal surface residue W418 of CYP11A1. Together, this indicates that cholesterol drives a conformational change toward the CYP11A1 proximal surface wherein reduced Adx M77 stabilizes the α helix-1 and -3 contacts. The mutant M77L led to a disruption of these contacts and resulted in changes focused towards α helix-2, suggesting an altered orientation of Adx M77L, which may contribute to a decrease in CYP11A1 activity.

An unexpected finding in this study was that the more drastic mutant M77S did not impair cholesterol metabolism but, surprisingly, enhanced vitamin D3 metabolism (Fig. 4*B*). NMR data revealed that Adx M77S also promoted line broadening towards α helix-1 with both substrates (Fig. 7*D*), whereas with WT Adx, this outcome was only observed with cholesterol. Considering that the NMR signal for Adx M77S α helix-3 is disrupted (Fig. 6), we theorize that replacement of methionine with the polar sidechain of serine may disrupt an internal Met-Leu contact with L80, thereby causing localized disorder on α helix-3 of Adx. It is remarkable that such a change either preserves CYP11A1 activity (as with cholesterol) or enhances activity (as with vitamin D3). This observation is consistent with the NMR structure of Adx in the reduced state, showing significant remodeling of α helix-3 (40). Therefore, we posit that Adx M77S may resemble structural features of reduced Adx, promoting functionally important steering interactions along α helix-1. In contrast, the Adx M77L mutant showed loss of α helix-1 broadening with cholesterol observed with WT, providing convincing evidence that interactions involving α helix-1 are in some way functionally important, despite the orientation of this region away from CYP11A1 in the co-crystal structure. Clearly, the functional role of Adx α helix-1 warrants further study.

Next, we mutated CYP11A1 corresponding residue W418A to elucidate its role in substrate metabolism. The W418A mutation on its own was not found to significantly disrupt either reaction (Fig. 5*A*). However, this mutant in combination with Adx M77L was able to restore WT levels of cholesterol metabolism that were previously lost with Adx M77L (Fig. 5*B*). Moreover, combining CYP11A1 W418A with Adx M77S nullified the increased vitamin D3 metabolism observed previously with WT CYP11A1 and Adx M77S, and did not further alter cholesterol metabolism (Fig. 5*C*). Together, these findings provide further evidence that Adx M77 and CYP11A1 W418 are a substrate-driven contact, as there are opposite effects on vitamin D3 monooxygenation and cholesterol side chain cleavage reactions, depending on the mutation.

In general, these data may relate the strength and nature of substrate binding of CYP11A1 to structural changes at the enzyme’s proximal surface. We theorize that cholesterol, which binds strongly in a type I manner, may induce conformational changes that enhance contacts between CYP11A1 W418 and the Adx M77. This may compensate for the W418A mutation, thereby not significantly affecting cholesterol metabolism. In contrast, vitamin D3 binds without inducing a strong type I response, with little net change in absorbance, which may result in fewer conformational changes at the proximal surface of CYP11A1. In this interpretation, the ability of W418A to restore cholesterol metabolism when combined with Adx M77L may be due to the increased hydrophobicity of the side chain, thus restoring a favorable interaction between CYP11A1 W418A–Adx M77L These findings provide further evidence in support of a revised model in which structural differences between cholesterol and vitamin D3 (and thus their binding modes) induce differing structural changes at the proximal surface, in turn altering CYP11A1 interactions with both α Adx helix-3 and secondary steering contacts with α Adx helix-1 in a substrate-specific manner.

A confounding element to this work is the increased vitamin D3 metabolism observed in the presence of Adx M77S. One possibility is that this mutation and the disorder at ⍺ helix-3 close the electron transfer distance, possibly allowing a new hydrogen-binding interaction between the serine alcohol and the W418 side-chain amide, while simultaneously weakening the affinity of the protein complex. A weakened affinity is evidenced by the decrease in net peak broadening observed in the presence of vitamin D3 (Fig. 7*A*). We note that methionine-aromatic interactions are known to contribute an additional 1 to 1.5 kcal/mol of binding energy in addition to hydrophobics (39). In comparison, M77S may significantly lessen the overall binding affinity, thus allowing Adx to dissociate more readily and re-enter the shuttling cycle, with the net effect being an increase in vitamin D3 metabolism.

While this study was focused on CYP11A1, it is likely that other CYPs also modulate function at this level, albeit in a CYP-specific manner. A sequence comparison of the mitochondrial CYPs that also metabolize some form of vitamin D3 shows a semi-conserved hydrophobic or aromatic residue that corresponds with W418 (2) (Sequence alignment is shown in Supporting Fig. S5). Interestingly, we were unable to dock rat CYP24A1 to reduced Adx when the corresponding proximal residue (I425, V425 in humans) and M77 were identified as an active interaction (data not shown). This is most likely due to the I425 sidechain facing away from the proximal surface and may explain why Adx M77L and M77S have a minimal effect CYP24A1 activity in comparison to CYP11A1 (Fig. 8*A*). The activity of the *Mycobacterial* enzyme CYP121 (containing an arginine in this position) was unaffected (Fig. 8*B*). These comparisons point toward what may be a CYP11A1 specific contact, although what role other more conserved residues, such as those found in the other five mitochondrial CYPs would need to be investigated. For example, CYP11B1, CYP11B2, and CYP27B1 feature a phenylalanine that corresponds with W418 of CYP11A1, while CYP27A1 and CYP27C1 feature a tyrosine and histidine, respectively. However, the crystal structures of CYP11B1, CYP11B2, and CYP11B2:Adx all in complex with fadrozole (PDB: 6M7X, 4FDH, and 7M8I, respectively) indicate that F418 is oriented away from the proximal surface where Adx interacts (9, 48, 49). Interestingly, the case of CYP11B1 and CYP11B2 provide an additional clinical rationale for understanding the CYP-specific contacts with M77 of Adx. A potential therapy for the treatment of Cushing disease is selective reduction in aldosterone *via* inhibition of CYP11B2 over the similar CYP11B1 (93% sequence identity) (8, 50). Such a strategy may benefit from mapping differences in substrate-driven CYP11B1 and CYP11B2 Adx interactions as we have performed here for CYP11A1.

Here we describe an NMR based protocol that we believe is generally applicable toward the investigation of other CYP enzymes, particularly those that metabolize steroids or secosteroids that can be adequately solubilized using HPCD. Moreover, this work also provides evidence that reactions mediated by CYP11A1 can be separated by a single surface contact between CYP11A1 and Adx, although the possibility that a corresponding regulatory site exists in other CYPs still warrants investigation.

## Experimental procedures

### Protein production

The plasmid encoding bovine CYP11A1 in a pCWori vector was a gift from the lab of Dr Irina Pikuleva. This construct of mature CYP11A1 consists of native residues 41 to 520 and contains a 4X histidine tag on the C-terminus. The plasmid was transformed into JM109 chemically competent *E. coli* (Promega) and selected for using 50 ug/ml carbenicillin. Site-directed mutagenesis was conducted on this plasmid through GenScript. Cultures were grown overnight (O/N) from a single colony in 125 ml of Luria Broth (LB) containing 50 ug/ml carbenicillin, and then 10 ml of the O/N culture was used to inoculate 500 ml of Terrific Broth media in 2.8 L Fernbach flasks. The cultures were grown at 37 °C with orbital shaking at 220 RPM until log phase growth (OD_600_ = 0.6–0.8) and then induced with 1 mM Isopropyl β-D-1-thiogalactopyranoside (IPTG) and 0.5 g/3L of 5-Aminolevulinic acid hydrochloride (ALA). Expression was carried out at 28–26 °C for 48 h with continued shaking at 200 RPM. Cells were harvested, and the pellets were stored as a paste at −80 °C until purification. After thawing the pellets, the cells were resuspended in 200 ml of lysis buffer containing 100 mM potassium phosphate (pH 7.4), 300 mM NaCl, 20% Glycerol, and 300 μL of ProBlock protease inhibitor cocktail (GoldBio). Cell lysis was initiated by the addition of 0.5 mg/ml lysozyme and 2 μg/ml DNase I (GoldBio) while stirring at 4 °C for 30 min. Solubilization was carried out with the addition of 1% CHAPS Detergent (3-((3-cholamidopropyl) dimethylammonio)-1-propanesulfonate) (GoldBio), with stirring at 4 °C for 30 min followed by sonication on ice in 30 s burst for a total time of 3 min using a Branson SFX Sonifier set to 60% amplitude. The solubilized protein was then subjected to ultracentrifugation (Beckman Coulter Ultra Optima XPN-100) at 104,000*g* for 1 h. The supernatant lysate was then loaded onto a nickel-nitrilotriacetic acid (Ni-NTA) column and washed with 10 column volumes of equilibration buffer A (100 mM potassium phosphate, 300 mM NaCl, 20% glycerol, 0.5% CHAPS, pH 7.4). The protein was eluted with a 0 to 100% buffer B gradient (100 mM potassium phosphate (pH 7.4), 300 mM NaCl, 20% glycerol, 0.5% CHAPS, 200 mM Imidazole) over 15 column volumes. The elution fractions with peak absorbances between 419 to 423 nm, indicating imidazole-bound CYP, were further purified by passage through a size exclusion chromatography column (Cytiva HiLoad 16/600 Superdex 75) using a buffer containing 100 mM potassium phosphate, 300 mM NaCl, 20% glycerol, 0.1% CHAPS, and pH 7.4 to reduce the final detergent concentration to 0.1%. The protein purity and stability were assessed through SDS-PAGE, absorbance ratio (417 nm/280 nm), and carbon monoxide assays.

Overexpression and purification of ^15^N-bovine Adx was conducted as previously described (27). Site-directed mutagenesis was conducted on this plasmid through GenScript. After utilizing a Ni-NTA as previously described, further purity was achieved by using a size exclusion chromatography column (Cytiva HiLoad 16/600 Superdex 75). Generation of CYP121 (51), rat CYP24A1 (34), and Adrenodoxin Reductase (28) was conducted as previously described.

### Substrate-binding assays

CYP11A1 spectral substrate binding assays were carried out using a Shimadzu UV-2700 spectrophotometer. Cholesterol and cholecalciferol (vitamin D3) titrations were done in the presence and absence of a 10-fold excess of Adx. Both substrates were incorporated into 25% HPCD (Cayman Chemicals). Each assay was performed in triplicate using 1 μM of CYP11A1 in assay buffer (50 mM potassium phosphate buffer, pH 7.4). A double-cuvette method was employed to reduce the signal contributed from substrates and HPCD. In this setup, 25%HPCD-choleseterol or -vitamin D3 was added to the sample cuvette containing 1 μM of CYP11A1, while the reference cuvette contained only 25%HPCD-substrate. For experiments involving excess Adx, the double-cuvette method was adjusted: Adx was added to both cuvettes before introducing CYP11A1 into the sample cuvette. To allow for complete binding, each substrate addition was followed by a 5-min incubation period before the acquisition of the spectrum. Affinity constants were calculated by fitting the substrate concentrations plotted against the difference spectra showing type-I response. The resulting plot was analyzed using Prism GraphPad software (version 7.05) and fitted using a single binding mode equation for hyperbolic binding as described previously (27). A time-course carbon monoxide (CO) assay was performed before and after each titration to assess the stability of CYP11A1 upon completion of substrate titrations. CO binding spectra were performed by first reducing samples of CYP11A1 with excess sodium dithionite, followed by bubbling of CO gas and immediately recording spectra between 400 nm and 500 nm at regular intervals of one scan every 120 s for 10 min.

### Reconstitution of functional assays

CYP11A1 reconstituted functional assays were carried out in 50 mM potassium phosphate, pH 7.4 at 37 °C for 45 and 90 min. The reaction volume was 90 uL, wherein 81uL of a master mix containing CYP11A1, substrate, Adx, and Adrenodoxin Reductase (AdR) was added to a well containing 9 uL of 10 mM NADPH to begin the reaction. Functional assays were conducted in two different methods, varying in CYP11A1 and Adx concentrations. The cholesterol side chain cleavage reactions included 2 μM CYP11A1, 50 μM 25% HPCD-cholesterol, 10 μM AdR, and 10 μM Adx, with the internal standard as 150 μM pregnenolone acetate (MP Biomedicals). The vitamin D3 monooxygenation reaction included 10 μM CYP11A1, 50 μM 25% HPCD-cholesterol (Alfa Aesar), 10 μM AdR, and 20 μM Adx, with 150 μM alfacalcidol (MedChemExpress) as the internal standard. The use of HPCD has previously been demonstrated to not alter the site of the reaction for both substrates (35, 36, 37). The reactions were quenched by addition of 180 uL of acetonitrile (AcN) followed by the addition of 10 uL of internal standard. Negative controls consisted of withholding either NADPH or the CYP. The quenched reaction mixture was then subjected to 30 min of centrifugation at 10,000*g*, and 100 uL of the supernatant was injected onto a reverse-phase high pressure liquid chromatography (Agilent 1220 LC) column (InfinityLab Poroshell 120 EC-C18). The CYP11A1 cholesterol reactions used a mobile phase consisting of 80% AcN in water and 0.1% formic acid (FA) with a flow rate of 0.5 ml/min at 25 °C. Elution peaks were detected by monitoring the absorbance at 205 nm. The formation of pregnenolone was quantified as a ratio of the internal standard. The CYP11A1 vitamin D3 reactions used a mobile phase of 100% AcN with a flow rate of 0.5 ml/min at 40 °C. Elution peaks were detected by monitoring the absorbance at 264 nm. The depletion of vitamin D3 was quantified as a ratio of the internal standard. To more directly compare CYP11A1 reactions that were measured using both product formation as well as substrate depletion, the relative change in CYP11A1 activity was calculated as a fold-change from WT. Briefly, the ratio of HPLC peak areas was normalized with the internal standard and compared to WT using the following equation; [*(mutant activity/WT activity) – 1*], with a zero value indicating no net change from the WT condition. The absolute value of the net change was then set to be above or below zero depending on whether it represented an increase or decrease in the relative activity of the reaction. The CYP121 phenol coupling reaction of CYP121 and the vitamin D3 hydroxylation reaction CYP24A1 were measured as described previously by our group (34, 52). Statistical analysis was carried out by one-way ANOVA or two-way ANOVA where significance indicated with a *p* value was less than 0.05. Post-hoc analysis of ANOVA significance was performed using Tukey’s or Dunnett’s test.

### 2D NMR data acquisition and analysis

All NMR samples contained 50 μM ^15^N-Adx for 2D ^1^H-^15^N HSQC acquisition in low salt buffer (50 mM potassium phosphate, 50 mM NaCl, pH 7.2). CYP11A1 was first washed with high salt buffer (300 mM potassium phosphate, 300 mM NaCl, 20% Glycerol, pH 7.2) in a 50 kDa cutoff concentrator (Millipore) to remove extraneous detergent. CYP11A1 was then transferred into a 10 kDa cutoff concentrator containing ^15^N-Adx prior to exchange into the final NMR buffer. Samples that contained substrates incorporated into 25%HPCD were added after buffer exchange into the pre-formed CYP11A1:Adx complex, followed by the addition of 5% deuterium oxide (ThermoScientific). Data acquisition and processing was carried out as previously described (Natalie 2024). NMRViewJ was utilized to extract the amide peak intensities of ^15^N-Adx. For spectra of ligand-free CYP11A1 on the Adx complex, the following equation was used: Intensity_CYP11A1+Adx_/Intensity_Adx_. The overall average intensity remaining and standard deviation was calculated, where peaks displaying peak attenuation of 1, 1.25, and 1.50 standard deviations away from the per residue average were mapped onto the crystal structure of Adx (PDB:1CJE) as red, purple, and blue, respectively. To assess the impact of substrates on the preformed CYP11A1–Adx complex, the following equation was used: Intensity_Substrate + CYP11A1+Adx_/Intensity_CYP11A1+Adx_. The overall average intensity remaining and standard deviation was calculated, where less or greater than one standard deviation away from the average were mapped onto the crystal structure of Adx as blue and green, respectively.

### Cytochrome C assay

To assess whether Adx mutations disrupt interaction with AdR, a Cytochrome C assay was performed by measuring absorbance changes in Cytochrome C at 550 nm and thus indirectly monitoring the reduction of Adx by AdR. The assay was conducted with 100 μM of Cytochrome C (MP Biomedical), 0.5 μM WT or mutant Adx, and 0.5 μM AdR in 200 uL final volume in 50 mM potassium phosphate (pH 7.4). The reaction was initiated by addition of 190 uL of mastermix into a 96-well plate containing 10 uL of NADPH and the change in absorbance recorded using a BioTek Cytation 5 Cell Imaging Multimode Reader. The experiment was conducted in triplicate and significance different relative to WT Adx was assess with Prism as mentioned previously.

### CYP11A1–Adx docking

Protein docking was carried out using HADDOCK (version 2.4) (44, 45). The docking was guided by the identification of key active residues, W418 in bovine CYP11A1 (PDB: 3MZS) and M77 in reduced bovine Adx (PDB: 1L6V), which were designated as interaction sites. HADDOCK 2.4 automatically performs a cluster and model analysis, and the resulting docked protein complex with the best HADDOCK score was visualized and illustrated using PyMOL software.

## Data availability

Raw and processed data from this study are stored digitally and are available upon request.

## Supporting information

This article contains supporting information.

## Conflict of interest

The authors declare that they have no conflicts of interest with the contents of this article.
