# Supplementary material for: NMR-guided identification of CYP11A1–Adrenodoxin interactions that differentially govern cholesterol and vitamin D3 metabolism
Source: J Biol Chem. 2025 Jun 26;301(8):110428. doi: 10.1016/j.jbc.2025.110428 (PMC12309602; doi:10.1016/j.jbc.2025.110428)
Supplement: Supporting information [file mmc1.docx]

**Supporting information:**

NMR-guided Identification of CYP11A1–Adrenodoxin Interactions that Differentially Govern Cholesterol and Vitamin D3 Metabolism

**Janie E. McGlohon^1^, Jacob Logothetis^1^, D. Fernando Estrada^1^***

1. Department of Biochemistry, Jacobs School of Medicine and Biomedical Science, University at Buffalo, Buffalo, New York, USA

*Corresponding author, email: dfestrad@buffalo.edu

**Running title:** Substrate-specific Modulation of the CYP11A1–Adx Interaction

**Supporting Figure S1. Intensity plot showing differential peak broadening of ^15^N-Adx upon titration of CYP11A1.** Titration of A) 12.5 μM, B) 25 μM, C) 37.5 μM, and D) 50 μM CYP11A1 against 50 μM ^15^N-Adx. The intensity plot illustrates the average (

𝜇) Adx peak intensity remaining (----, black) resulting from the interaction with CYP11A1. A cutoff of 1 standard deviation (----, red) away from the average

(

𝜇 – 1 S.D.) was applied to identify residues that undergo peak broadening relative to the overall assigned peaks, marked by a loss of peak intensity ratio greater than 1.50 (blue), 1.25 (purple), or 1.0 (red) standard deviation away from the average remaining intensities. Amide peaks near the 2Fe-2S cluster are not observable.

**Supporting Figure S2. Intensity plot showing differential peak broadening of the ^15^N-Adx:CYP11A1 (1:0.5) complex upon titration of substrates.** The intensity plot shows HPCD (gray), substrate (pink), and substrate-specific (yellow) effects on ^15^N-WT Adx peak intensity in the presence of CYP11A1 and 25%HPCD A) -empty, B) -cholesterol, and C) -vitamin D3. Differentially affected residues are marked by a decrease (blue) or increase (green) of peak intensity ratio greater than 1.0 standard deviation (----, red) away from the average remaining intensities (----, black).


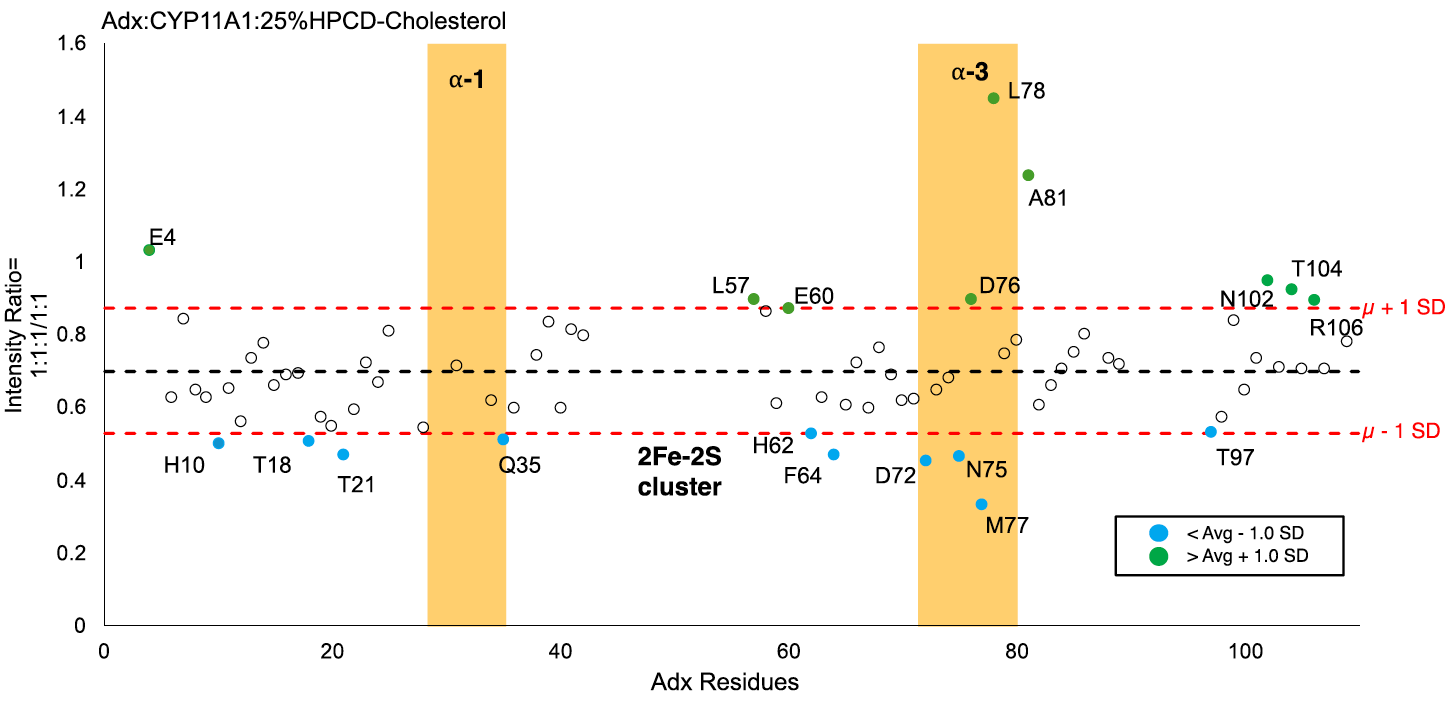


**Supporting Figure S3. Intensity plot showing differential peak broadening of the ^15^N-Adx:CYP11A1 (1:1) complex upon addition of cholesterol.** Differentially affected residues are marked by a decrease (blue) or increase (green) of peak intensity ratio greater than 1.0 standard deviation (----, red) away from the average remaining intensities (----, black).

**Supporting Figure S4. Cholesterol binding to CYP11A1 with and without Adx present.** Titration of cholesterol against 1 μM A) CYP11A1 (black), and in the presence of excess (10 μM) Adx B) WT (blue), C) M77L (red), and D) M77S (orange). Titration were run in triplicate with errors bars indicating one standard deviation, and binding curves measured the absorbance difference between ~390 nm and ~420 nm. The corresponding difference spectra of cholesterol binding to CYP11A1 in the absence and presence of excess Adx are directly below (E-H).


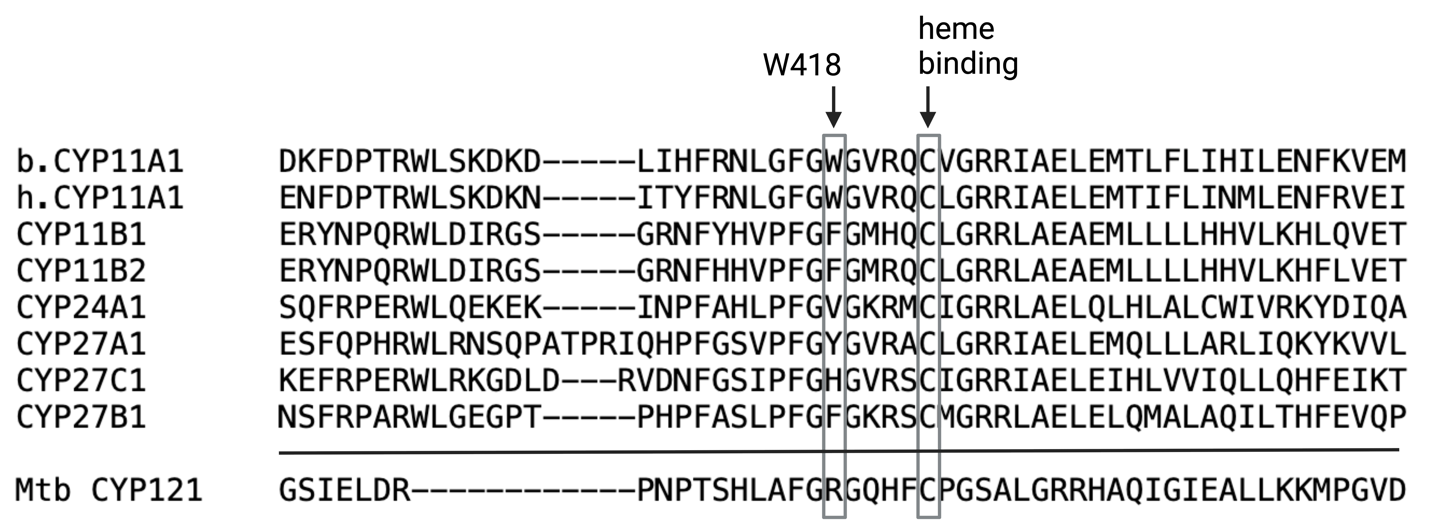


**Supporting Figure S5**. Sequence alignment of the proximal surface of vitamin D metabolizing CYPs. Both bovine and human CYP11A1 are displayed. All other sequences are human. CYP121 of *Mycobacterium tuberculosis* does not metabolize vitamin D but is shown for comparison.
